# The Use of Antibiotic Prophylaxis

**DOI:** 10.1155/S1064744997000458

**Published:** 1997

**Authors:** Sebastian Faro


Infectious Diseases in Obstetrics and Gynecology 5:271 (1997)
© 1998 Wiley-Liss, Inc.

### The Use of Antibiotic Prophylaxis

Antibiotic prophylaxis has afforded the surgeon an advantage to operate in a potentially contaminated field and, in most instances, allowed the patient to escape the morbidity of infection. However, the surgeon has taken the liberty to use antibiotics for prophylaxis in operative procedures for which such a use has not been substantiated. This reliance on antibiotic prophylaxis for procedures other than cesarean section, vaginal hysterectomy, abdominal hysterectomy, and elective abortion has not been proven to be efficacious.

Antibiotic prophylaxis is used for a wide variety of gynecologic procedures for which there are no data to support its use. This indiscriminate use has even spread to the use of antibiotic combinations.

Use of antibiotic prophylaxis has expanded to result in not only exposing bacteria to a large amount of the agent, but also an increased variety of antimicrobial agents. This liberal use of antibiotics is exerting significant selective pressure on the patient's bacterial population as well as the hospital bacterial population. The selection of resistant strains of bacteria is a serious problem that has made treatment more difficult. Although there has been much written and discussed about the selection of resistant bacteria, it appears to be falling on deaf ears. Gynecologists and obstetricians continue to administer antibiotics with little concern for their ability to select resistant strains under the pretense that it is preventing postoperative infectious morbidity. It would appear that the various groups of gynecologic surgeons, e.g. laparoscopists, pelvic surgeons, urogynecologists, and oncologists could perform collaborative studies to investigate the role of antibiotic prophylaxis in various operative procedures. These studies are urgently needed to determine if antibiotic prophylaxis is needed and to help curb the indiscriminate use of antibiotics. These studies are also needed to reduce the selection of resistant strains and help reduce the costs to the patients.

*Sebastian Faro, MD, PhD*
*Editor-in-Chief*

